# Quercetin Inhibits Lef1 and Resensitizes Docetaxel-Resistant Breast Cancer Cells

**DOI:** 10.3390/molecules25112576

**Published:** 2020-06-01

**Authors:** Marta Prieto-Vila, Iwao Shimomura, Akiko Kogure, Wataru Usuba, Ryou-u Takahashi, Takahiro Ochiya, Yusuke Yamamoto

**Affiliations:** 1 Division of Cellular Signaling, National Cancer Center Research Institute, Tokyo 104-0045, Japan; mprietov@tokyo-med.ac.jp (M.P.-V.); ishimomu@ncc.go.jp (I.S.); akogure@tokyo-med.ac.jp (A.K.); w2usuba@marianna-u.ac.jp (W.U.); tochiya@tokyo-med.ac.jp (T.O.); 2 Department of Molecular and Cellular Medicine, Institute of Medical Science, Tokyo Medical University, Tokyo 160-0023, Japan; 3 Department of Urology, St. Marianna University School of Medicine, Kanagawa 216-8511, Japan; 4 Department of Cellular and Molecular Biology, Hiroshima University, Hiroshima 734-8553, Japan; rytakaha@hiroshima-u.ac.jp

**Keywords:** quercetin, drug resistance, Lef1, breast cancer

## Abstract

Drug resistance is a major problem for breast cancer patients. Docetaxel is an anti-mitotic agent that serves as first line of treatment in metastatic breast cancer, however it is susceptible to cellular drug resistance. Drug-resistant cells are able to spread during treatment, leading to treatment failure and eventually metastasis, which remains the main cause for cancer-associated death. In previous studies, we used single-cell technologies and identified a set of genes that exhibit increased expression in drug-resistant cells, and they are mainly regulated by Lef1. Furthermore, upregulating Lef1 in parental cells caused them to become drug resistant. Therefore, we hypothesized that inhibiting Lef1 could resensitize cells to docetaxel. Here, we confirmed that Lef1 inhibition, especially on treatment with the small molecule quercetin, decreased the expression of Lef1 and resensitized cells to docetaxel. Our results demonstrate that Lef1 inhibition also downregulated ABCG2, Vim, and Cav1 expression and equally decreased Smad-dependent TGF-β signaling pathway activation. Likewise, these two molecules worked in a synergetic manner, greatly reducing the viability of drug-resistant cells. Prior studies in phase I clinical trials have already shown that quercetin can be safely administered to patients. Therefore, the use of quercetin as an adjuvant treatment in addition to docetaxel for the treatment of breast cancer may be a promising therapeutic approach.

## 1. Introduction

Drug resistance to anticancer therapies is a major problem worldwide. Drug resistance leads to treatment failure and tumor recurrence and eventually to metastasis, which dramatically decreases the 5-year survival rate of breast cancer patients despite a good initial prognosis [1]. Docetaxel (DTX) is an anti-mitotic agent that serves as a standard adjunct treatment in breast cancer, and is used as first line of treatment in metastatic breast cancer. Its use has been associated with a statistically significant reduction in mortality; however, it is susceptible to cellular drug resistance caused, for instance by drug efflux-associated proteins [2]. Previously, we used single-cell technology and performed in depth analysis on the dynamic alterations of the transcriptome in a luminal subtype of breast cancer cells, MCF7 cells, throughout the acquisition of drug resistance to DTX [3]. In this study, we elucidated a set of genes responsible for drug resistance. The identified drug-resistant gene profile included an increase of epithelial-to-mesenchymal transition (EMT) and stemness-related genes and a decrease of cell cycle regulators genes. We further confirmed that their regulation was mainly controlled by the transcription factor Lef1 and that, under *LEF1* overexpression conditions, the expression of the other genes was upregulated to ultimately increase the resistance to DTX.

Lymphoid enhancer-binding factor-1 (Lef1) is a transcription factor that plays a central role in the Wnt signaling pathway. Generally, after its activation, β-catenin binds to the LEF/TCF complex to promote the transcription of multiple molecules related to stemness [4,5]. Lef1 has also been shown to act independently of β-catenin, both during embryogenesis and cancer [6,7]. Lef1 is highly expressed in some cancers, such as colon, ovarian cancer, and leukemia [8,9,10], where it has been reported to be associated with poor prognosis and to be essential for cancer invasion and metastasis [9,11,12]. However, Lef1 overexpression has not been described as a common phenomenon in Luminal subtype of breast cancer tissue; according to our prior data less than 10% of tumors presented high amount of Lef1 expressing cells. Nonetheless, we recently showed that Lef1 is expressed in a small subpopulation of breast cancer cells in half of tumor samples, providing them with an elevated drug resistance [3]. Lef1 overexpression in MCF7 parental cells increased their IC_50_ to DTX 4.5 folds by the consequent upregulation of ATP-binding cassette sub-family G member 2 (ABCG2), Vimentin (Vim), and transforming growth factor-beta 2 (TGF-β) signaling pathway activation [3].

**Figure 1:**
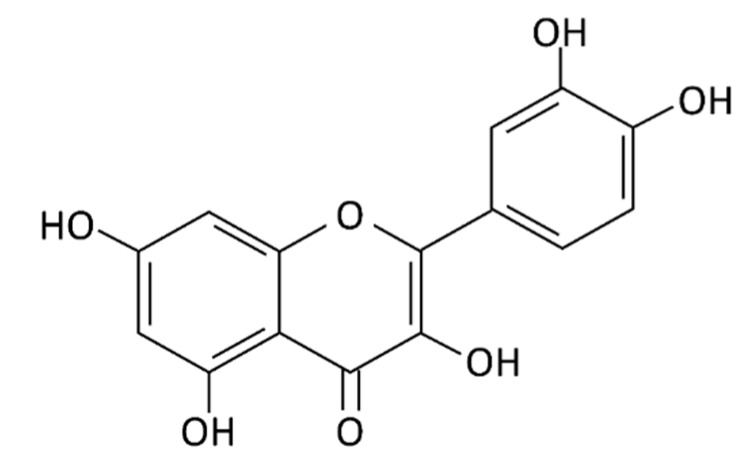
Chemical structure of quercetin.

Taking into consideration that the overexpression of *LEF1* induced DTX resistance in parental MCF7 cells, we hypothesized that the depletion of Lef1 might resensitize resistant cells to DTX. With this objective, we selected the small molecule, quercetin (Figure 1), which has been previously described to inhibit the Wnt signaling pathway; more concretely, quercetin inhibits Lef1 pathway transcriptional activity [13]. Quercetin treatment inhibited cell growth in cell lines from cancer types where Lef1 expression was high, such as colon and ovarian cancer cells [13,14]. Here, we show that following quercetin treatment, Lef1 was downregulated, with a consecutive downregulation of Caveolin 1 (Cav1), ABCG2, Vim and TGF-β signaling pathway inactivation. Together, these molecules resensitized drug-resistant cells, presenting a synergetic effect with DTX. 

## 2. Results

### 2.1. LEF1 Inhibition Regulates the Drug Resistance-Associated Gene Expression Profile

**Figure 2:**
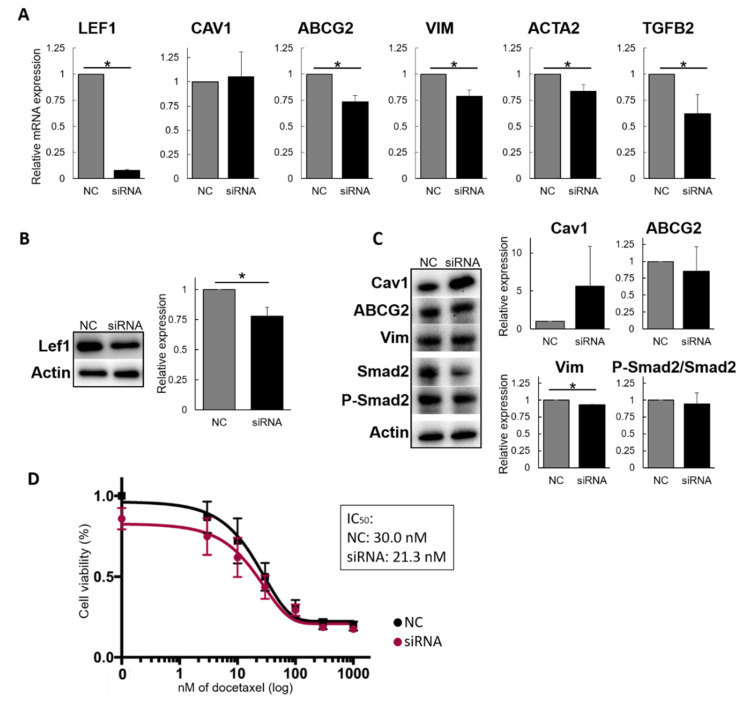
Effect of LEF1 siRNA on drug resistance. (**A**) Gene expression alterations induced by *LEF1* knockdown by siRNA. (**B**) Representative Western blot showing Lef1 levels after siRNA treatment. The right graph shows the quantitation of the levels (*n* = 3). (**C**) Representative Western blot showing the protein levels responsible for drug resistance acquisition (Left panel). Averaged relative amount of protein expression of Cav1, ABCG2 and Vim after *LEF1* siRNA treatment, and ration between P- Smad2 and total Smad2 representing Smad-dependent TGF-β signaling pathway activation (Right panel) (*n* = 3). (**D**) Proliferation assay of MCF7-DR cells with Docetaxel (DTX) after siRNA treatment. The graph represents the mean of 4 individual experiments. IC_50_ values are as follows: NC = 30 ± 13.3 nM; Lef1 siRNA = 21.3 ± 11.7 nM (*n* = 5); *p*-value = 0.026.

Since Lef1 overexpression induced an associated change in gene expression that eventually conferred DTX drug resistance, we evaluated whether the inhibition of Lef1 could revert this resistance. First, *LEF1* mRNA expression was knocked down in MCF7-DR cells by treatment with a pool of siRNAs. There was significantly decreased *LEF1* expression at 72 h after transfection (Figure 2A). Moreover, changes in gene expression in treated cells affected not only *LEF1* but also other genes, such as *ABCG2, VIM, Actin alpha-2 (ACTA2),* and *TGFB2* (Figure 2A); the expression of *CAV1* was not changed. Subsequently, the effect of *LEF1* siRNA treatment was analyzed at the protein level by Western blot (Figure 2B). While mRNA levels were 10-times lower in treated cells, protein levels were reduced by only 20%. The low inhibition of Lef1 at protein level, lead to even a lower decrease of protein levels of ABCG2, Vim and Phosphorilated-Smad2(P-Smad2)/Smad2 ratio. While the protein expression of all the molecules was slightly decreased, the difference with negative control was not significant, except for Vim (*p =* 0.0086) (Figure 2C). Next, the effect of *LEF1* mRNA depletion on DTX resistance was investigated. Loss of *LEF1* resulted in reduced viability even without the presence of DTX, and this was maintained until high levels of DTX were added (Figure 2D). The IC_50_ significantly decreased from 30 ± 13.3 nM in NC to 21.3 ± 11.7 nM in siRNA- treated cells, indicating that inhibition of *LEF1* mRNA levels decreases cell viability to some extent.

### 2.2. Quercetin Inhibits Lef1 Protein in a Dose-Dependent Manner and Resensitizes DTX-Resistant Cells

Next, due to the recent popularity of small molecules, we searched the literature for molecules involved in the Wnt signaling pathway, looking specifically for a Lef1 inhibitor. Quercetin (Figure 1), a well-known flavonoid, was selected since it had been shown to regulate the transcriptional activity of the Lef1 signaling pathway [13]. The effect of quercetin on Lef1 was corroborated by adding quercetin at low levels of (1–40 μM) to avoid excessive cell death, and then the mRNA and protein expression levels of several genes were analyzed. 

**Figure 3:**
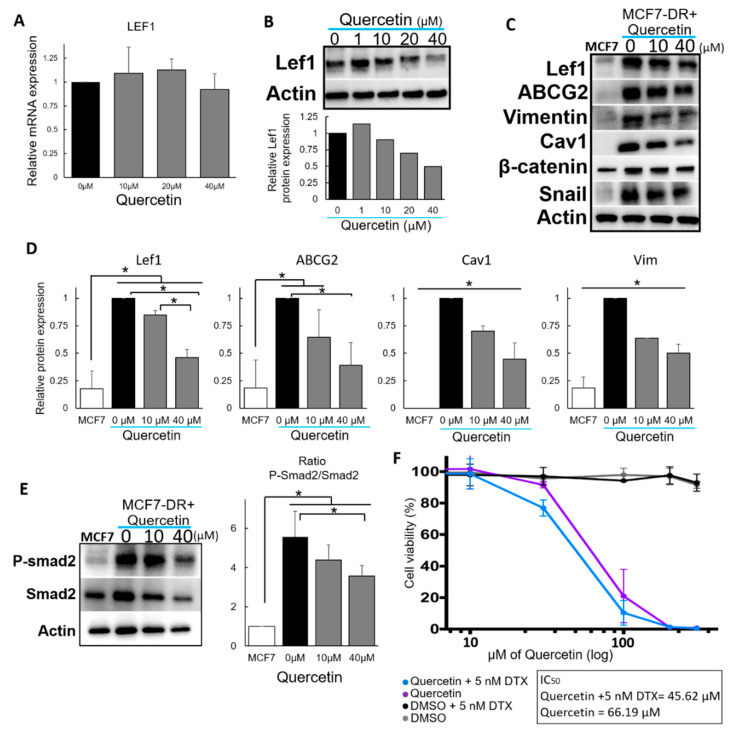
Effect of quercetin on drug resistance. (**A**) *LEF1* mRNA levels for cells treated with several different concentrations of quercetin. (**B**) Lef1 protein levels for cells treated with several different concentrations of quercetin, and their quantification (lower part) (*n* = 1). (**C**) Representative images of Western blot showing ABCG2, Vim, Cav1, Snail and β-catenin protein levels in MCD7-DR cells 3 days after quercetin treatment (*n* = 3). (**D**) Relative protein expression of Lef1, ABCG2, Cav1 and Vim in MCD7-DR cells 3 days after quercetin treatment. Lef1 MCF7-DR 0 μM vs. MCF7-DR 10 μM and 40 μM *p*-value = 0.077 and >0.001 respectively; ABCG2 MCF7-DR 0 μM vs. MCF7-DR 10 μM and 40 μM *p*-value = 0.053 and >0.001 respectively; Cav1 MCF7-DR 0 μM vs. MCF7-DR 10 μM and 40 μM *p*-value = >0.001 in both cases; Vim MCF7-DR 0 μM vs. MCF7-DR 10 μM and 40 μM *p*-value = >0.001 in both cases (*n* = 3). (**E**) Western blot image of Smad2 and P-Smad2 after quercetin treatment. The right panel shows the ratio of P-Smad2 and Smad2, representing Smad-dependent TGF-β signaling pathway activation, MCF7 vs. MCF7-DR (0 μM, 10 μM and 40 μM) *p*-value = 0.0007, 0.004 and 0.018 respectively; quercetin treatment 0 μM vs. 40 μM *p*-value = 0.049. (**F**) Proliferation assay results for MCF7-DR cells treated under several conditions to assess the effect of quercetin. The graph represents the mean of 4 individual experiments. IC_50_ are as follows: quercetin +5 nM DTX = 45.62 ± 6.4 µM; querceti*n* = 66.19 ± 15.9 µM. DMSO with and without 5 nM DTX did not show any significant effect on cell viability (*n* = 4); *p*-value = 0.034.

Although the addition of quercetin did not affect *LEF1* mRNA levels (Figure 3A), Western blot results showed a strong decrease in Lef1 expression in a dose-dependent manner (Figure 3B). Furthermore, Lef1 inhibition was accompanied by the downregulation of ABCG2, Vimentin, and Cav1 in MCF7-DR cells (Figure 3C,D). A decrease in Smad-dependent TGF-β signaling pathway activity was also indicated by the reduced ratio of P-Smad2/Smad2 (Figure 3E) and the downregulation on the TGF-β signaling downstream, Zinc finger protein SNAI1 (Snail) (Figure 3C) [15]. However, similar to our previous results [3], the depletion of Lef1 did not affect β-catenin protein expression (Figure 3C).

Finally, we analyzed the effect of quercetin on MCF7-DR, a resistant cell line. The addition of quercetin alone showed a moderate cytotoxic effect on MCF7-DR cells, as indicated by their IC_50_ of 64.8 µM. Furthermore, when cells were treated with quercetin in addition to 5 nM DTX, the viability of MCF7-DR cells was shown to be affected at a dose that had an IC_50_ 1.5-fold lower than that of quercetin alone (Figure 3F). This difference suggested an additive or synergetic effect between quercetin and DTX. 

### 2.3. Quercetin and DTX Have a Synergistic Effect

**Figure 4:**
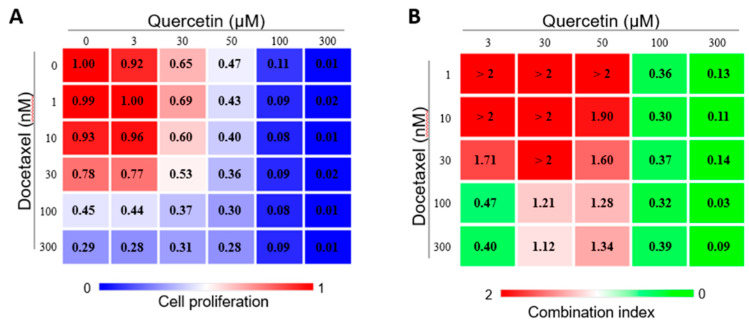
Quercetin presents a synergetic effect to DTX. Combinatorial experiment of quercetin and docetaxel. (**A**) Left panel shows the cell viability rate, (**B**) and right panel shows the combinatory index. CI >1 indicated antagonism, CI = 1 additive and CI <1 synergistic effect (*n* = 3).

To further analyze the potential synergetic effect between quercetin and DTX on the downregulation of Lef1 to resensitize cells, we calculated the Chou–Talalay combination index [16]. Figure 4A shows the cell viability under several concentrations of quercetin and DTX in combined treatment; Figure 4B shows the combinatory index. Levels that are lower than 1 indicate a synergetic effect of the two components. Middle-to-high levels of quercetin (from 100 µM), which are represented in light green colors, showed a high synergetic effect with even low doses of DTX (Figure 4B).

Taken together, the results indicate that quercetin treatment on drug-resistant cells inhibited Lef1 protein expression, which subsequently downregulated the protein expression of ABCG2, Vim, and Cav1 and decreased Smad-dependent TGF-β signaling pathway activation. The depletion of these proteins eventually resensitized cells to DTX.

## 3. Discussion

Lef1 is a DNA-binding transcription factor that has a central role in the canonical Wnt signaling pathway. Aberrant expression of Lef1 has been associated with poor prognosis in several cancers [10,11,12,17,18,19]. In addition to these reports, we recently found that the induction of Lef1 expression in the luminal A subtype of breast cancer cells increased their resistance to DTX. In this study, we found that Lef1 inhibition, especially by treatment with the small molecule quercetin in drug-resistant cells, re-established their sensitivity to DTX. 

Aberrant Lef1 expression has been widely associated with poor prognosis in leukemia [10,17] and several solid cancers such as colorectal [18,19], lung adenocarcinoma [12], and endometrial carcinoma [8]. Although Lef1 expression has not been found to be significant in breast cancer [20], our laboratory has recently reported a novel role for Lef1 in breast cancer drug resistance [3]. Indeed, Lef1 expressing cell amount was negligible, and close to 0 in the luminal A subtype of breast cancer cell parental lines MCF7 and ZR-75 cells. However, both cell lines showed a great increase in Lef1 expression upon DTX resistance acquisition. As a transcription factor of the Wnt signaling pathway, Lef1 regulates the expression of several stemness-related molecules [4,5]. In addition, it is a facilitator of EMT induction [17,21], both by the downregulation of the epithelial markers [7] and the upregulation of mesenchymal marker proteins [6,22]. Upregulation of Lef1 in breast cancer parental cells induced an upregulation of a combination of proteins, eventually inducing drug resistance [3]. Thus, we hypothesized that Lef1 could be a very interesting target for reducing DTX resistance.

In our first experimental strategy, we attempted to reduce Lef1 expression by using a *LEF1* siRNA. Despite the fact that the *LEF1* mRNA levels were downregulated and the IC_50_ was reduced, this reduction in drug resistance may not be significant enough for potential use in clinical applications. The moderate effect of *LEF1* siRNA treatment on DTX resensitization was probably due to the low level of Lef1 protein reduction. Moreover, the discrete reduce in Lef1, resulted also in a very low decrease in its downstream protein expression, including ABCG2 and Vim. One possible reason for this could be the moderate half-life of Lef1 [23] and the difficulty of siRNA to eliminate proteins with long half-life times [24,25]. To overcome this problem, we decided to investigate the effects of small molecules, which could inhibit Lef1 at the protein level to exhort a stronger effect on docetaxel drug resistant cells. The use of small-molecule inhibitors has been increasing exponentially. These compounds, usually smaller than 500 kDa, can translocate directly through the plasma membrane and interact with multiple molecules within the cells. For this study, quercetin was selected. Quercetin is the most common flavonoid in nature and has recently drawn much attention because it has many biological effects, including antioxidant [26] and anti-inflammatory activity though NF-κB pathway inhibition [27]. Importantly, quercetin is known to induce cancer cell death by targeting the Wnt signaling pathway and specifically affecting the transcription of the pathway [13]. Its efficacy has been demonstrated in leukemia, colon, colorectal, and ovarian cancer [13,14,28,29], all of which are well known for high Lef1 expression [9,10,17,18,19]. Similar to previous reports, when we treated drug-resistant cells with quercetin, the Lef1 protein level decreased in a dose-dependent manner, and the effect on Lef1 was much greater than that seen with the siRNA treatment. Additionally, quercetin inhibited the protein expression of ABCG2, Vim, and Cav1, and it reduced Smad-dependent TGF-β signaling pathway activity. The reduction in these proteins collectively resensitized the cells to DTX; indicating a synergetic effect of quercetin with DTX. Notably, quercetin has been reported to be harmless to healthy cells [30]. Moreover, in 1996, a phase I clinical trial with quercetin was conducted, and no significant toxicities were confirmed until reaching a dose of 1700 mg/m^2^ [31]. Thus, the use of quercetin is a feasible and rapidly available approach. Due to the synergetic effects of quercetin with DTX and the low toxicity for patients, we propose quercetin to be a potential novel therapy to be used with DTX treatment.

Nevertheless, we cannot discard the possibility that the high effect of quercetin on DTX resistance may be due to an off-target effect. It is tempting to speculate that quercetin targets not only Lef1 but also other molecules, which eventually repress the protein expression of the set of genes responsible for drug resistance. One example for this would be Cav1. The Cav1 levels did not increase under *LEF1* overexpression and did not decrease in response to *LEF1* knockdown, indicating a likely indirect regulation by Lef1. However, its correlation with Lef1 at mRNA, and protein in vitro and in patient tissue samples was very high and constant [3]. Moreover, its expression decreased under quercetin treatment, suggesting an indirect result. In fact, this is not the first time that quercetin has been described to decrease Cav1 expression levels [32,33]. It is also known that quercetin targets other signaling pathways such as NF-κB and Pi3k/Akt/mTOR signaling pathway [34,35]. For instance in prostate cancer, where the Pi3k/Akt/mTOR signaling pathway plays a pivotal role in DXT resistance [36], quercetin treatment decreased both Akt and p-Akt protein expression and induced apoptosis. A combinatory treatment of quercetin with DTX was especially effective against DTX-resistant prostate cancer cells through the Pi3k/Akt/mTOR signaling pathway inhibition [37]. Combinations between DTX, quercetin and other molecules such as green tea phenols have also been reported to resensitize DR prostate cancer cells [38,39]. However, it is still not clear whether this is the main route of effect of quercetin in breast cancer. The Pi3k/Akt/mTOR pathway, a central protein synthesis regulator, is linked to the Wnt signaling pathway since Akt inactivates GSK-3 eventually leading to β-catenin accumulation [40]. Therefore, an inactivation of Pi3k/Akt/mTOR signaling pathway by quercetin would originate in a change of β-catenin protein amount. In our experiments, we did not observe any effect of quercetin on β-catenin expression. This is supported by the fact that we had not observed a change of mRNA levels of molecules from the Pi3k/Akt/mTOR pathway during drug resistance acquisition [3] suggesting that the Pi3k/Akt/mTOR pathway does not play a key role in our DTX resistance model. Moreover, since quercetin treatment did not affect β-catenin, the Lef1 upstream, nor mRNA levels of *LEF1*, it strongly suggests that quercetin interacts with Lef1 protein. Previous papers also rejected the possibility of quercetin interacting with β-catenin, since its expression was not altered; moreover quercetin reduced the biding of Tcf4, a member of LEF/TCF transcription factor family, to Tcf response elements (TRE) [13]. To date, the exact mechanisms of Lef1 inhibition by quercetin are not known. While it has been reported that quercetin, and other flavonoids containing catechol can act as TATA-binding protein (TBP)-like protein inhibitors [23], the concrete interaction mechanism of quercetin with Lef1, Pi3k, or other target molecules remains unclear and is an interesting topic to approach.

Our results suggested that Lef1 is regulated by the TGF-β signaling pathway rather than β- catenin. As previously mentioned, neither Lef1 expression changes by Lef1 overexpression nor downregulation by quercetin affected β-catenin levels. This idea of Lef1 being independent of β- catenin is not new. Kobayashi et al. reported that a mutant *LEF1* lacking the binding region could still exhibit EMT in cancer cells, and the same result was seen in cells without the β-catenin gene [6,41]. Indeed, Lef1 expression can be induced by other signals, such as TGF-β, which during embryogenesis is phosphorylated by Smad2 and Smad4 [7]. In our previous experiments, when Lef1 was overexpressed, it also induced increased TGF-β signaling pathway activation, suggesting a relation between these two pathways. While there are no previous reports connecting EMT and quercetin in cancer, quercetin prevented EMT by regulating the Smad2/3 phosphorylation pathway in retinal epithelial cells [42]. In our model of drug-resistant cells, quercetin also decreased the expression of TGF-β, emphasizing the relationship between Lef1 and TGF-β, although this hypothesis should be confirmed in future experiments. Thus, we propose quercetin as a potential novel drug to be used with DTX as a therapy, since we observed a synergetic effect between the two in drug- resistant cells in vitro. 

## 4. Materials and Methods 

### 4.1. Cell Culture and Drug Resistance Generation

MCF7 cells were purchased from American Type Culture Collection (ATCC, Manassas, VA, USA) and tested to ensure that they were mycoplasma contamination-free. An MCF7 cell line that is resistant to 5 nM DTX (MCF7-DR) was established as previously described [3]. Both cell lines were maintained with RPMI-1640, which was supplemented with 10% FBS and 1× antibiotics and antimitotic (Gibco, Grand Island, NY, LA, USA) in the case of MCF7 and 2 μg/mL of puromycin (Gibco, Grand Island, NY, USA) and 5 nM of DTX (Sanofi Aventis, Paris, France) in the case of MCF7-DR. In all cases, cells were grown at 37 °C in a 5% CO_2_ incubator, and the medium was changed every 3 days; passaging was performed when confluence reached 70–80%.

### 4.2. Lef1 siRNA Transfection and Real-Time PCR

MCF7-DR cells were plated in 6-well plates at a density of 1 × 10^5^ cells per well. The following day, SmartPool LEF1 siRNA (#51176) or nontargeting pool siRNA (Dharmacon, Lafayette, CO, USA) was added to cells with Lipofectamine RNAiMAX Reagent (Invitrogen, Carlsbad, CA, USA) according to the manufacturer’s instructions. Twelve hours after the transfection, the medium was changed, and the cells were incubated for 60 more hours. Total RNA was extracted from cell lines using QIAzol reagent and the miRNeasy Mini Kit (Qiagen, Venlo, Netherlands) according to the manufacturer’s instructions. Reverse transcription from 1 μg of total RNA was carried out using a High Capacity cDNA Reverse Transcription Kit (Applied Biosystems, Waltham, MA, USA). Real-time PCR analysis was performed using TaqMan Universal PCR Master Mix (Applied Biosystems, Waltham, MA, USA) on a StepOne Real-Time PCR System (Applied Biosciences, Waltham, MA, USA). The TaqMan probes used in this study were *LEF1* (Hs01547250_m1), *CAV1* (Hs00971716_m1), *ABCG2* (Hs01053790_m1), *VIM* (Hs00958111_m1), *ACTA2* (Hs00426835_g1), *TGFB2* (Hs00234244_m1) and *ACTB* (Hs03023880_g1). The expression levels were normalized to those of *ACTB*, and the relative fold changes in mRNA expression levels were calculated using the formula 2^−ΔΔCt^.

### 4.3. Quercetin Treatment

For Lef1 inhibition with the small molecule, quercetin (Sigma, Kawasaki, Japan) was diluted in DMSO to a 150 mM stock solution and filtered. The chemical structure of quercetin is represented in Figure 1. For mRNA and protein extraction purposes, MCF7-DR cells were seeded in 6-well plates at a density of 1 × 10^5^ cells. The following day, different concentrations of quercetin (range from 1 μM to 40 μM) were added to a medium without DTX, and cells were cultured for three more days. 

### 4.4. Proliferation Assay

MCF7-DR cells were seeded in 96-well plates at a density of 5000 cells per well. For siRNA inhibition studies, the following day, SmartPool LEF1 siRNA (#51176) or Nontargeting pool siRNA (Dharmacon, Lafayette, CO, USA) were added to cells with Lipofectamine RNAiMAX Reagent (Invitrogen, Carlsbad, CA, USA) according to the manufacturer’s instructions. Eight hours after the transfection, the medium was changed, and different concentrations of DTX (ranging from 3 nM to 1000 nM) were added to the new medium, which was followed by incubation for 2 days. For quercetin studies, on the day after plating the cells, normal medium containing 5 nM DTX was changed to different concentrations of quercetin (ranging from 3 μM to 300 μM) with or without the addition of 5 nM DTX, and the cells were incubated for 72 more hours. For the Chou–Talalay combination index, on the day after plating cells, the medium was changed to different concentrations of quercetin (ranging from 3 μM to 300 μM) and DTX (ranging from 0.3 nM to 300 nM), and the cells were incubated for 72 h. After the appropriate treatment, the supernatant was removed, and new medium containing 10% Cell Counting Kit-8 reagent (Dojindo Molecular Technologies, Kumamoto, Japan) was added. After 2 h of incubation at 37 °C, absorbance at 450 nm was measured using a Synergy H4 Microplate Reader (BioTek, Winooski, VT, USA). Viability of treated cells was calculated relative to the viability of untreated cells, and the IC_50_ was calculated using ImageJ software, while graphs were drawn using the curve fitting analysis tool in Prism 7 (GraphPad Software, San Diego, CA, USA).

### 4.5. Western Blotting

Cells were lysed to extract protein using Mammalian Protein Extract Reagent (Thermo Scientific, Rockford, IL, USA), and the concentration was measured using a Qubit (Invitrogen, Carlsbad, CA, USA). Ten micrograms of total protein sample was separated using SDS-PAGE gradient gels (Mini-Protean TGX Gel, GE Healthcare, IL, USA) and transferred onto PVDF membranes (Millipore, Burlington, MA, USA). After blocking, the membranes were incubated overnight at 4 °C with primary antibodies against Lef1 (#sc-374522, Santa Cruz, CA, USA), Vimentin (#5741S, Cell Signaling Technologies, Danvers, MA, USA), Caveolin-1 (#3267S, Cell Signaling Technologies, MA, USA), ABCG2 (#4278S, Cell Signaling Technologies, Danvers, MA, USA), β-catenin (#2698S, Cell Signaling Technologies, Danvers, MA, USA), Smad2 (#5339S Cell Signaling Technologies, Danvers, MA, USA), Phospho-Smad2 (#18338S, Cell Signaling Technologies, Danvers, MA, USA), Snail (#3895S, Cell Signaling Technologies, Danvers, MA, USA) and Actin C4 (#mab1501, Millipore, Burlington, MA, USA). Secondary antibodies linked to horseradish peroxidase anti-mouse IgG (NA931, GE Healthcare, Chicago, IL, USA) or anti-rabbit IgG (#NA934, GE Healthcare, Chicago, IL, USA) were incubated with the membranes for one more hour. Signals were developed using ImmunoStar LD (Wako, Osaka, Japan), and imaging and subsequent quantification were performed using Fusion Solo software with a Fusion Solo S microscopy imaging system (Vilber, Vilber Lourmat, France). To calculate the ratio of activated Smad2, after normalizing against Actin, the levels of phosphorylated-Smad2 were divided into those of Smad2.

### 4.6. Chou–Talalay Combination Index

The combinatory effects of drugs were analyzed by the proliferation assay as described in the proliferation assay section, using an 8 × 8 matrix format to assess 8 different concentrations in three independent experiments. The average matrix was then, screened to 6 concentrations to ensure the removal of unreliable data with a r > 0.95, as previously described [16]. After analyzing the proliferation assay, the Chou–Talalay combination index (CI) method was calculated using CompuSyn software as described [16,43] to assess the synergetic effect. CI > 1 indicated antagonism, CI = 1 indicated an additive effect and CI < 1 indicated a synergetic effect.

### 4.7. Statistical Analysis

All the data are expressed as the mean ± SD. A two-tailed Student’s *t*-test or the Holm–Bonferroni method were used to determine significant differences in the data. The results were considered statistically significant when the *p*-value was < 0.05.
